# Distributions of the National Early Warning Score (NEWS) across a healthcare system following a large-scale roll-out

**DOI:** 10.1136/emermed-2018-208140

**Published:** 2019-03-06

**Authors:** Lauren J Scott, Niamh M Redmond, Joanna Garrett, Penny Whiting, Kate Northstone, Anne Pullyblank

**Affiliations:** 1 NIHR CLAHRC West, University Hospitals Bristol NHS Foundation Trust, Bristol, UK; 2 Population Health Sciences, University of Bristol, Bristol, UK; 3 West of England Academic Health Science Network, Bristol, UK; 4 Department of General Surgery, North Bristol NHS Trust, Bristol, UK

**Keywords:** clinical assessment, emergency care systems, prehospital care, communications, emergency ambulance systems emergency department

## Abstract

**Background:**

Early warning scores (EWS) were developed in acute hospital settings to improve recognition and response to patient deterioration. In 2012, the UK Royal College of Physicians developed the National Early Warning Score (NEWS) to standardise EWS across the NHS. Its use was also recommended outside acute hospital settings; however, there is limited information about NEWS in these settings. From March 2015, NEWS was implemented across the healthcare system in the West of England, with the aim that NEWS would be calculated for all patients prior to referral into acute care.

**Aim:**

To describe the distribution and use of NEWS in out-of-hospital settings for patients with acute illness or long-term conditions, following system wide implementation.

**Method:**

Anonymised data were obtained from 115 030 emergency department (ED) attendances, 1 137 734 ambulance electronic records, 31 063 community attendances and 15 160 general practitioner (GP) referrals into secondary care, in the West of England. Descriptive statistics are presented.

**Results:**

Most attendance records had NEWS=0–2: 80% in ED, 67% of ambulance attendances and 72% in the community. In contrast, only 8%, 18% and 11% of attendances had NEWS ≥5 (the trigger for escalation of care in-hospital), respectively. Referrals by a GP had higher NEWS on average (46% NEWS=0–2 and 30% NEWS ≥5). By April 2016, the use of NEWS was reasonably stable in ED, ambulance and community populations, and still increasing for GP referrals.

**Conclusions:**

NEWS ≥5 occurred in less than 20% of ED, ambulance and community populations studied and 30% of GP referrals. This suggests that in most out-of-hospital settings studied, high scores are reasonably uncommon.

**Key messages**

**What is already known on this subject**

- The National Early Warning Score (NEWS) will be mandated for use in hospital by April 2019.
- NEWS is also recommended for use in out-of-hospital settings.
- Little is known about the distributions of NEWS in out-of-hospital settings and whether higher scores are associated with worse outcomes.

**What this study adds**

- Distributions of NEWS in out-of-hospital settings are presented for the first time.
- NEWS ≥5 occurred in less than 20% of emergency department, ambulance and community populations studied.
- These data should go some way to reassure healthcare professionals who were worried that many patients may have high NEWS regardless of acute illness.

## Introduction

Early Warning Scores (EWS) are widely recommended for recognising patients at risk of deterioration.^1^ Scores are calculated from a series of physiological observations, with higher scores indicating a patient is more unwell. In the UK, a number of different EWS systems have been used, mostly in hospital settings.^2–4^ To address the limitations of using a variety of different scores across the NHS, the Royal College of Physicians (RCP) developed the National Early Warning Score (NEWS), which was introduced in 2012 with inconsistent adoption.^5 6^ NEWS is a simple scoring system of six physiological measurements: respiratory rate, oxygen saturation, temperature, systolic blood pressure, heart rate and level of consciousness. Each measure is scored from 0 to 3 and added together to give an overall score with an additional two points for supplemental oxygen. Scores lie between 0 and 20, with higher scores resulting from worse physiological measurements (online supplementary appendix A). In secondary care, escalation triggers are scores of 3, 5 and 7, with three triggering hourly observations if there is a weighting of three points within a single parameter, five triggering hourly observations (regardless of weighting) and seven triggering a critical care referral; in the current system, these levels of care can only be delivered in-hospital.

10.1136/emermed-2018-208140.supp1 Supplementary file 1

The early detection of changes in physiological parameters provides an opportunity to initiate a timely and competent clinical response and improve outcomes.^5 7–12^ Historically, EWS have been used in secondary care settings, but NEWS has been advocated by the RCP for use in out-of-hospital settings in the UK,^5^ such as general practice, mental health services and ambulance services and also by the UK Royal College of Emergency Medicine to detect sepsis.^1 13–15^

In 2015, the West of England Academic Health Science Network (AHSN) introduced NEWS to all healthcare settings across the region. The aim was to use NEWS for prompt recognition of severe illness and as a standard communication tool for acutely unwell patients to support escalation from the community into acute care. For consistency, the thresholds for action were aligned to those already used in secondary care (3: threat, 5: refer and 7: severe).

In order to deliver this large-scale project, the Institute for Healthcare Improvement breakthrough collaborative model was used^16^: representatives from all sectors came together every 6 months to share progress and exchange ideas. Collaborative event discussions and results of a formal qualitative evaluation^17^ have produced some common themes. First, that NEWS is not validated for use in an out-of-hospital environment, and second, that use of NEWS might increase secondary care referrals as there could be patients in the community with high NEWS (eg, NEWS ≥5) where the clinical opinion is that referral is not required. Analysing pragmatic real-life data from out-of-hospital settings, in this study, may help to address the validity of these concerns. Similarly, there is apprehension about using NEWS for patients with long-term conditions as they might have high ‘baseline’ NEWS due to their condition meaning that, while using NEWS to monitor changes over time would be useful, an absolute score might not. For example, patients with chronic obstructive pulmonary disease (COPD) often have low oxygen saturation and/or need supplemental oxygen (both components of NEWS).

Despite its recommended use outside of secondary care settings, there is limited data on NEWS in these settings to inform implementation. Therefore, the aim of this study is to describe the distribution of NEWS among acutely and chronically unwell populations in out-of-hospital settings using large West of England datasets. In addition, we will describe changes in its use over time following the roll-out across the healthcare system. As a secondary analysis, we will describe the relationship between NEWS and length of hospital stay and mortality in the emergency department (ED).

## Methods

### Introduction of NEWS

The introduction of NEWS into all healthcare settings in the West of England started in March 2015. An initial launch event engaged organisations from acute, urgent, community and primary care and mental health services and commissioners from across the region; the event covered five clinical commissioning areas of: Bristol, North Somerset and South Gloucestershire; Bath and North-East Somerset; Swindon; Wiltshire; and Gloucestershire.

In order to facilitate data collection, the West of England AHSN funded the introduction of NEWS into the South Western Ambulance Service electronic patient care record and the development of templates into IT systems such as Adastra, EMIS and SystemOne used by primary and community services. Hospitals receiving general practitioner (GP) calls via a single point of access changed IT systems to record handover NEWS electronically during referral.

### Data collection and manipulation

For the purposes of this descriptive study, retrospective anonymised routinely collected data were obtained from four providers of healthcare in different parts of the system. Data were accessed by NHS staff (who had permission to access the data and were not associated with this study) and provided to the researchers anonymised. Within each dataset, if multiple scores were recorded within one attendance, only the first score was reported. Data were retrospectively collected back until April 2016, 1 year following the start of the roll-out.

1. A total of 115 030 ED attendances to North Bristol NHS Trust (NBT), a large suburban acute trust with a major trauma centre (April 2016–August 2017).
2. A total of 1 137 734 South Western Ambulance Service NHS Foundation Trust (SWASfT) attendances, the sole provider of emergency ambulance services across the South West (April 2016–November 2017).
3. A total of 31 063 visits to patients being cared for by Bristol Community Health (BCH), the leading provider of community services within Bristol Clinical Commissioning Group area (April 2016–September 2017). Data from BCH only included attendances where NEWS was calculated during this time.
4. A total of 15 160 referrals to the General Practice Support Team (GPST), a call centre manned by GPs that receives all referrals from primary care into NBT ED (April 2016–August 2017).

Patients using ED and ambulance services represent self-reported acutely unwell patients; patients using community services are chronically unwell or have acute exacerbations; and patients referred to GPST are acutely unwell as diagnosed by a GP.

For datasets 1, 2 and 3, vital signs data were entered into IT systems, and NEWS were automatically calculated; NEWS could only be calculated if patients had a complete set of observations. For dataset 4, there was no such IT system in place; NEWS may have been calculated using the app, other systems or manually.

For the purpose of this paper, NEWS from all four data sources were grouped into six categories: 0, 1–2, 3–4, 5–6, 7–8 and 9+.

Patients under the age of 16 years have been removed from the GPST dataset and are not seen by the BCH service so they do not appear in the data; in NBT ED and SWASfT, NEWS is not calculated for patients <16 years, so they are reported in the ‘no NEWS recorded’ group. Similarly, NEWS is not calculated for pregnant women (or patients who do not consent to observations) and such patients will also appear in the no NEWS group (and not appear at all in the BCH dataset).

Attendances to NBT ED were grouped into majors, minors and resus depending on where they were sent following triage. ED protocols for minor patients state that NEWS is only required for patients with minor illness, head injuries or rib injuries. The remainder of patients in minors will have minor injuries (eg, a broken wrist) and do not require NEWS to be calculated as deterioration is rare; this patient cohort accounts for a large proportion of those seen in minors. Very sick patients in majors and resus who were rushed straight through to treatment or surgery did not have NEWS calculated. Additionally, some patients had NEWS recorded on paper but not entered onto the database. All these patients are therefore included in the ‘No NEWS recorded’ category.

No NEWS was recorded for a 12-day period in October 2016 in the NBT ED dataset, which is assumed to be a data/database glitch. As we only had aggregated monthly numbers of the attendances without NEWS, we have excluded all October 2016 data from any NBT ED analyses to remove any bias caused by the missing data.

Missing data are described (and referred to as ‘No NEWS recorded’), but no imputation has been performed as this is a purely descriptive analysis. As data were provided anonymised, we do not know if the same patient accessed services on multiple occasions. As such, we referred to each score belonging to an attendance rather than a patient.

### Data analysis

Stata V.15.1 was used to conduct all data checking, cleaning and analyses. Descriptive statistics—means, SD, medians, IQR, ranges, counts and percentages—were used to analyse the data.

## Results

**Figure 1:**
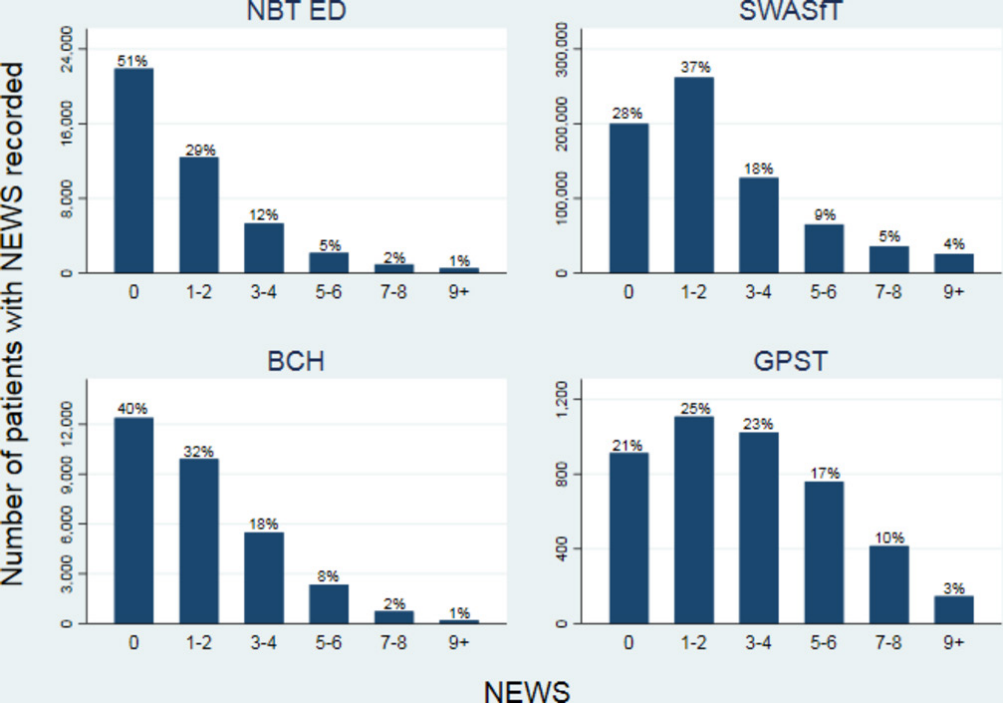
Distributions of NEWS. Note. Not all percentages add up to 100% due to rounding. BCH, Bristol Community Health; ED, Emergency Department; GPST, General Practice Support Team; NBT, North Bristol NHS Trust; NEWS, National Early Warning Score; SWASfT, South Western Ambulance Service NHS Foundation Trust.

**Figure 2:**
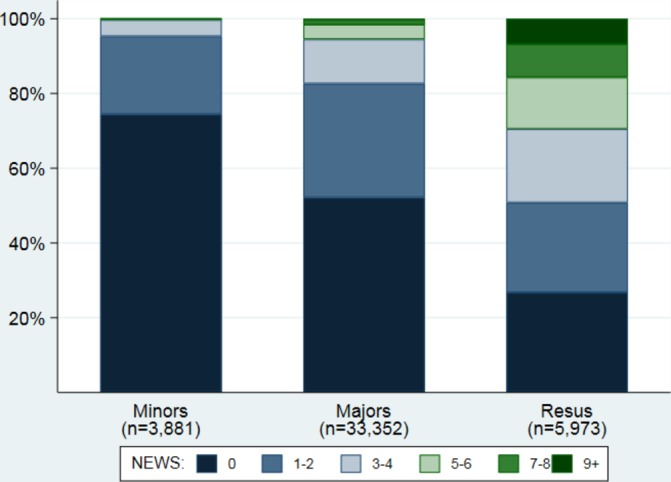
Distribution of NEWS on admission to the emergency department by admission stream. NEWS, National Early Warning Score.

Of 115 030 attendances to NBT ED between April 2016 and August 2017 (mean age 56 years, SD 23), 38% had NEWS recorded at triage (66% of majors, 7% of minors and 57% of resus attendances; table 1). The median recorded NEWS was 0, IQR 0–2 and range 0–17; only 8% had NEWS ≥5 (figure 1); this proportion was greater in resus than majors or minors (29%, 5% and <1%, respectively; figure 2). The percentage of attendances who had NEWS recorded changed very little over time; 39% and 38% of attendances had a score recorded in April 2016 and August 2017, respectively.

**Table 1:** Percentage of patients with NEWS recorded over time

Month	ED minors		ED majors		ED resus		SWASfT		GPST		BCH COPD	BCH unplanned	BCH planned
	n	%	n	%	n	%	n	%	n	%	n	n	n
April 16	270/3404	7.9	2066/2879	71.8	370/636	58.2	23 120/38 685	59.8	100/881	11.4	84	924	382
May 16	245/3535	6.9	2214/3113	71.1	395/699	56.5	24 226/40 195	60.3	124/811	15.3	78	953	476
June 16	234/3291	7.1	2121/3125	67.9	394/694	56.8	23 467/38 991	60.2	171/844	20.3	85	778	546
July 16	295/3809	7.7	2153/3194	67.4	372/658	56.5	27 870/45 733	60.9	131/896	14.6	63	1010	593
August 16	226/3510	6.4	2062/3075	67.1	429/695	61.7	29 630/48 705	60.8	135/920	14.7	112	903	619
September 16	201/3600	5.6	1751/3073	57.0	357/678	52.7	30 566/49 974	61.2	171/909	18.8	90	864	550
October 16	–	–	–	–	–	–	34 439/56 785	60.6	290/877	33.1	93	950	646
November 16	251/3109	8.1	1982/3001	66.0	379/648	58.5	35 521/59 378	59.8	271/847	32.0	131	1182	785
December 16	271/2973	9.1	2204/3284	67.1	398/668	59.6	40 499/64 947	62.4	299/815	36.7	186	1128	781
January 17	254/2857	8.9	2074/3237	64.1	340/585	58.1	41 365/64 732	63.9	370/983	37.6	230	1307	750
February 17	202/2754	7.3	1964/2884	68.1	309/539	57.3	36 551/57 507	63.6	313/791	39.6	216	1018	709
March 17	230/3393	6.8	2205/3230	68.3	331/599	55.3	39 623/62 946	62.9	330/882	37.4	176	955	728
April 17	249/3512	7.1	2193/3256	67.4	363/668	54.3	38 714/60 943	63.5	280/718	39.0	167	859	594
May 17	264/3663	7.2	2174/3403	63.9	394/698	56.4	40 760/63 922	63.8	322/839	38.4	103	888	729
June 17	236/3658	6.5	2067/3226	64.1	365/643	56.8	39 805/61 824	64.4	323/832	38.8	109	927	699
July 17	197/3594	5.5	2044/3412	59.9	388/655	59.2	42 024/64 493	65.2	374/826	45.3	107	902	661
August 17	256/3274	7.8	2078/3295	63.1	389/644	60.4	41 964/63 150	66.5	362/811	44.6	83	906	630
September 17	–	–	–	–	–	–	40 577/62 462	65.0	–	–	86	957	575
October 17	–	–	–	–	–	–	43 047/66 514	64.7	–	–	–	–	–
November 17	–	–	–	–	–	–	42 053/65 848	63.9	–	–	–	–	–

BCH, Bristol Community Health; COPD, chronic obstructive pulmonary disease; ED, Emergency Department; GPST, General Practice Support Team; NEWS, National Early Warning Score; SWASfT, South Western Ambulance Service NHS Foundation Trust.

Of 1 137 734 attendances by SWASfT between April 2016 and November 2017, 63% had NEWS recorded. The median NEWS was 1, IQR 0–3, range 0–20; 18% had NEWS ≥5 (figure 1). The percentage of attendances with NEWS recorded changed very little over time; 60% and 64% of attendances had a score recorded in April 2016 and November 2017, respectively (table 1).

**Figure 3:**
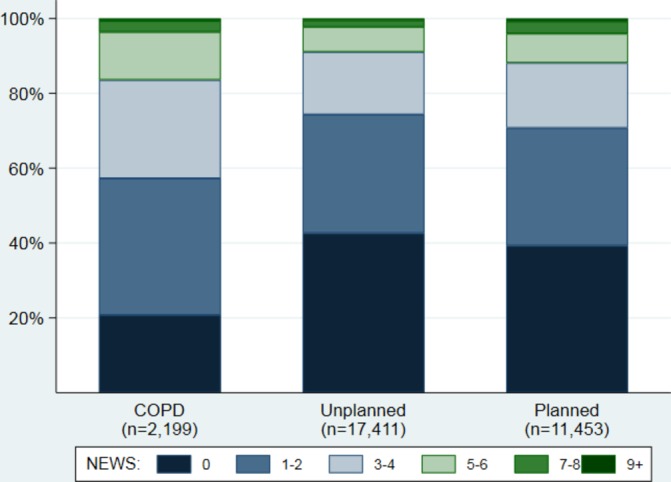
Distribution of NEWS in the community by service. Chronic obstructive pulmonary disease (‘COPD’) includes patients who are regularly monitored in the community for their COPD. ‘ Unplanned’ includes acutely unwell patients. ‘ Planned’ includes patients who have recently been discharged from hospital and/or have chronic conditions in need of monitoring. NEWS,  National Early Warning Score.

The 31 063 attendances in the community by BCH with NEWS recorded had a median score of 1, IQR 0–3 and range 0–20. Overall, 11% of attendances had NEWS ≥5 (figure 1): 17% in the COPD service, 9% of unplanned visits and 13% of planned visits (figure 3). The percentage of attendances in the COPD service with NEWS of 0 (21%) was far lower than in other services (43% unplanned and 39% planned). The number of attendances with NEWS recorded across all services increased by 16% during the time period; 1390 and 1618 attendances had a score recorded in April 2016 and September 2017, respectively (table 1).

In the GPST service (mean age 61 years, SD 22), 4366/14 482 (30%) attendances had NEWS recorded. The median score was 3, IQR 1–5, range 0–14; 30% had NEWS ≥5% and 13% had NEWS ≥7 (figure 1). The percentage of attendances with NEWS recorded increased over time, with only 11% reporting a score in April 2016 compared with 45% in August 2017 (table 1).

**Figure 4:**
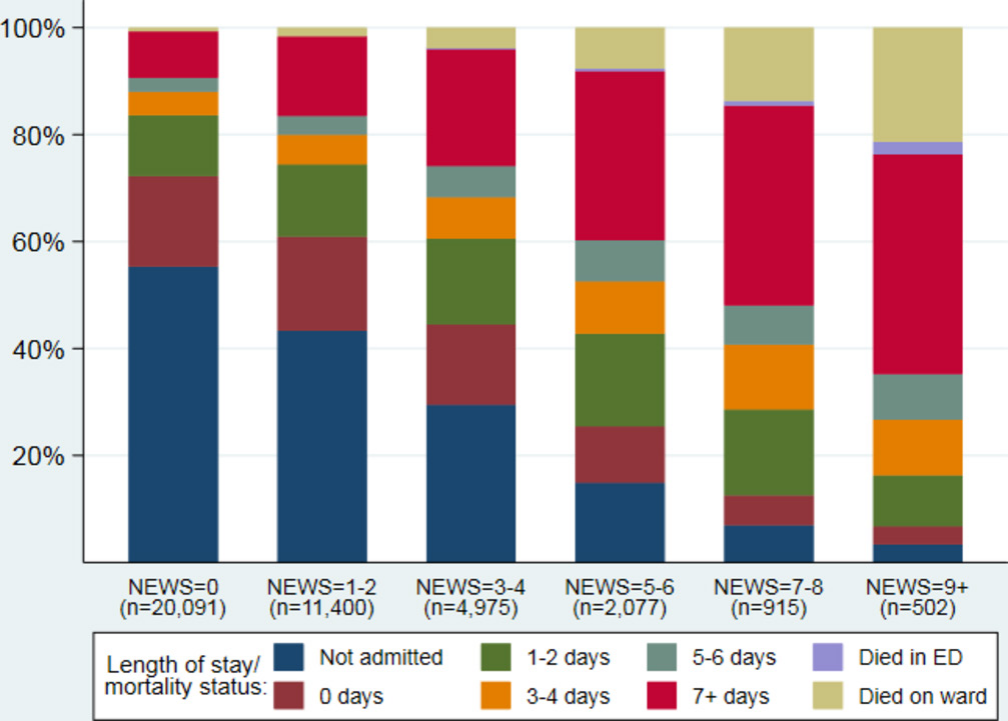
Length of hospital stay/mortality status by NEWS on admission to ED. ED, Emergency Department; NEWS, National Early Warning Score.

On average, attendances where higher NEWS was recorded at ED triage were more likely to be admitted, have longer lengths of stay and higher mortality (figure 4) than attendances with lower NEWS recorded. For example, the proportion of attendances who were admitted to hospital or died in ED was far higher in those with NEWS ≥7 (94%) than those with NEWS=0 (45%).

## Discussion

### Summary of results

This is the first description of the distributions and use of NEWS in out-of-hospital settings following a system-wide roll-out and adoption. The key finding was that, in attendances where NEWS was recorded, NEWS ≥5 occurred in less than 20% of attendances to ED, by the ambulance service or the community. The numbers of scores recorded in each setting over time suggested that NEWS was adopted sooner in ED and the ambulance service than by community services and GPST. By April 2016 (approximately 12 months after the start of the roll-out), the use of NEWS appeared to be reasonably stable in ED, ambulance and community populations, with use in GPST continuing to rise due to slow adoption; work to raise awareness in primary care is ongoing. Additionally, our secondary analysis of the ED data showed attendances with higher NEWS at triage were more likely to be admitted, have longer lengths of hospital stay and higher mortality than attendances with low NEWS, suggesting NEWS is a suitable tool in this setting.

### Strengths and limitations

The main strengths lie in the large pragmatic data available from several different sources and the similar distributions of NEWS across ED, the ambulance service and community health. These data have not previously been published and provide some reassurance about using NEWS in out-of-hospital settings. Concerns have been raised that many people in the community who are well may have a high ‘baseline’ NEWS; however, our data suggest that even among patients with long-term illnesses (BCH data), only 11% had NEWS ≥5.

Most of the limitations pertain to data quality. Patients at NBT ED who were very sick (and would therefore probably have higher NEWS) may not have had a score calculated at triage due to needing urgent and immediate treatment, or because clinicians could see they require emergency care without needing to measure all NEWS components. Additionally, the data provided by GPST included all notes from the consultation, including NEWS, within a free-text box. Scores have been extracted by searching for common terms and extracting numerical data, meaning it is possible that some scores may have been missed, and some spurious scores may have been included. Furthermore, the BCH data did not include patients for whom NEWS was not recorded so we were unable to assess how often NEWS was used in this setting in terms of percentages. Reports from BCH suggest they have been slower to adopt NEWS than NBT ED and SWASfT; for example, in the COPD service, 34% and 39% of patients had NEWS recorded in 2016 and 2017, respectively (data not shown). Furthermore, as all presented data were collected routinely, they may contain data entry errors; we do not have component scores for any of the data sources so we were unable to check the accuracy of the data. However, as most scores were calculated using verified IT algorithms, this should not be major concern. As data were provided anonymously, it is likely that within one episode a patient may appear in multiple datasets. Although it is unfortunate that we could not track patients between services, we believe reporting the first score recorded by these four services is still of major interest to healthcare providers working within these fields. Finally, within the scope of this paper, we were unable to investigate whether the thresholds for action currently used both in hospital and out of hospital are appropriate. We recommend that further research is undertaken to investigate these.

### Comparison with existing literature

NEWS ≥5 is the recommended trigger for recognition of sepsis^15^ and is the score chosen to trigger a referral or discussion with secondary care. Our qualitative research^17^and discussions at collaborative events have found that there is a perception that NEWS ≥5 may be a common finding in patients without acute illness as well as patients with acute illness. Our key finding that this occurs in less than 20% of attendances to ED by the ambulance service and in the community disputes this perception.

In our recent systematic review of EWS in prehospital settings,^18^ all but one of the identified studies were based on ambulance service data with the other conducted in a nursing home. The ambulance service data in the review show that EWS have good accuracy for prediction of death within 48 hours. In addition, patients with EWS=0 were very unlikely to deteriorate and higher scores meant patients were more likely to deteriorate. Our ED data support these findings with only 0.03% of attendances with NEWS=0 dying within 48 hours, compared with 7.2% of attendances with NEWS ≥7. None of the NEWS papers included in the review described the distributions of NEWS among their patients so we are unable to compare them to our data.

Two studies published in 2018 investigating NEWS in the ambulance service did share their NEWS distributions.^19 20^ The first study^19 ^presented data on all patients based in two hospital districts in Northern Finland seen by the ambulance service over a 6-month period in 2014. They reported a median prehospital (ambulance) NEWS of 2 with a range of 0–18 and stated that 4342/12 426 (34.9%) patients had NEWS=0. This is similar to the NEWS distribution in our SWASfT ambulance data, despite the study being based in Northern Finland. The second study^20^ was much smaller including only 189 patients attended by the ambulance service and admitted to Royal London Hospital over a 20-day period in 2013. They reported a median prehospital NEWS of 3 and IQR 1–5. This is higher than our ambulance data but, as it only includes patients admitted to hospital, this is not surprising. In fact, the NEWS distribution in this small study is very similar to our GPST data, which includes a much more similar patient population.

Furthermore, two studies exploring NEWS in the ED also shared their NEWS distributions. In 2015, Alam *et al* 21 presented data collected over a 6-week period from an ED in Amsterdam that included 274 medium risk patients (scoring 2 or 3 on the Emergency Severity Index^22^). They reported NEWS at triage as median 2, IQR 1–4 and range 0–11; this population has a higher average NEWS than our ED majors population, which contains similar patients: median=0, IQR=0–2, range=0–15. In 2016, Bilben *et al* 23 presented data collected on 246 patients in respiratory distress over a 5-month period from an ED in Norway. They reported NEWS at triage as median 5 and IQR 3–7; this is again higher than our comparable resus population (median=2 and IQR=0–5).

As far as we are aware, there are no UK studies presenting NEWS distributions in the ED, community or a GP referral service, and as such data from our study will be of great interest to all healthcare professionals in these areas.

### Clinical implications for practice

The distributions of NEWS reported in this paper can provide reassurance to healthcare professionals who believe there are many patients who have high baseline NEWS regardless of acute illness. Furthermore, following concerns surrounding respiratory conditions such as COPD, NEWS2^15^ has recently been launched with a separate oxygen saturation subscale for such patients. Our data suggest that high NEWS (eg, ≥5 or ≥7) are uncommon, indicating perceptions about NEWS increasing referrals to secondary care could be unwarranted. Following the guidance for referral used in secondary care, in conjunction with clinical judgement and knowledge, is sensible and is unlikely to result in many unnecessary referrals.

## Conclusions

NEWS ≥5 occurred in less than 20% of ED, ambulance and community populations studied and 30% of attendances referred into secondary care by a GP. This suggests that in most out-of-hospital settings studied, high scores are reasonably uncommon. These data can address reported health professional’s concerns that use of NEWS in community settings may increase unnecessary referrals to secondary care.
