# Supplementary material for: Distributions of the National Early Warning Score (NEWS) across a healthcare system following a large-scale roll-out
Source: Emerg Med J. 2019 Mar 6;36(5):287–92. doi: 10.1136/emermed-2018-208140 (PMC6580766; doi:10.1136/emermed-2018-208140)

Appendix A: NEWS score card

National Early Warning Score (NEWS)

PHYSIOLOGICAL PARAMETERS	3	2	1	0	1	2	3
Respiration Rate	≤8		9 - 11	12 - 20		21 - 24	≥25
Oxygen Saturations	≤91	92 - 93	94 - 95	≥96			
Any Supplemental Oxygen		Yes		No			
Temperature	≤35.0		35.1 - 36.0	36.1 - 38.0	38.1 - 39.0	≥39.1	
Systolic BP	≤90	91 - 100	101 - 110	111 - 219			≥220
Heart Rate	≤40		41 - 50	51 - 90	91 - 110	111 - 130	≥131
Level of Consciousness				A			V, P, or U

West of England Academic Health Science Network

Royal College of Physicians

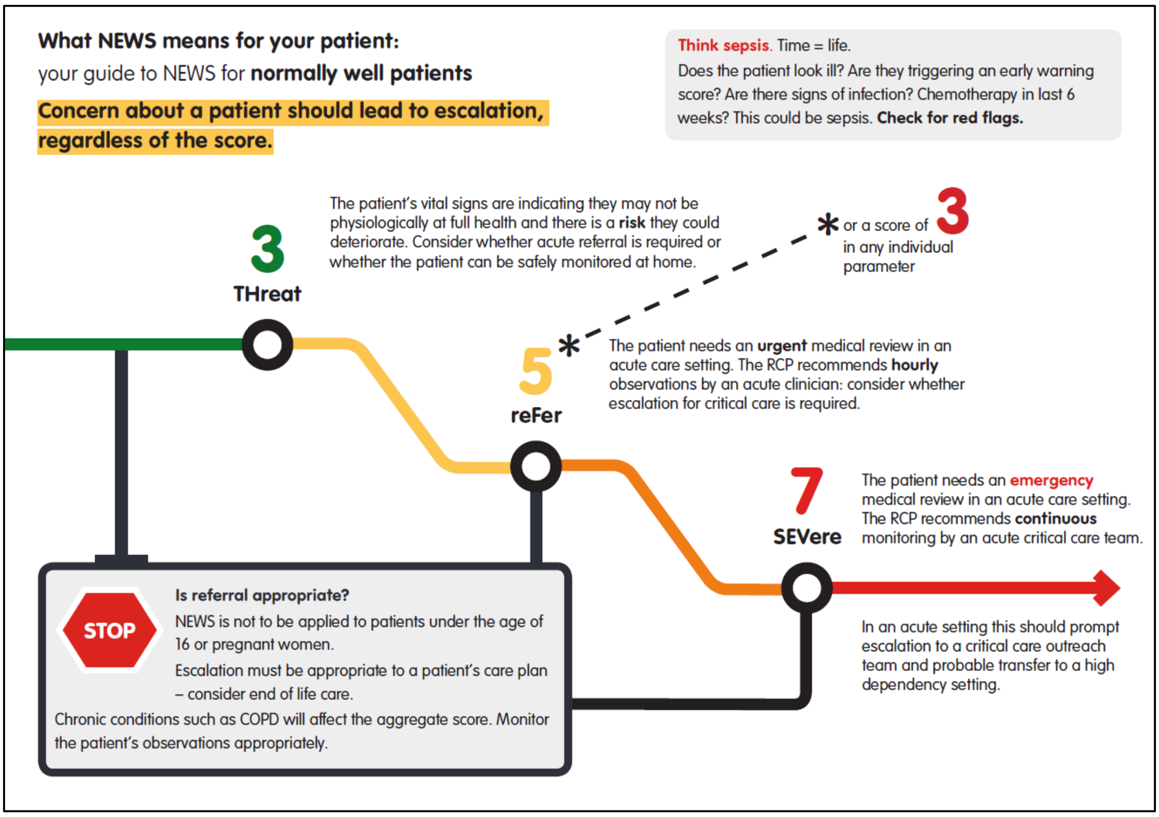

Supplement: Supplementary file 1 [file emermed-2018-208140supp001.pdf]
